# The Microscope in Medicine

**Published:** 1891-01-31

**Authors:** Frank J. Wethered


### The Microscope in Medicine.

By Frank J. Wethered, M.D.

### III.—The Microtome and Hardening Specimens.

Specimens which have to be examined microscopically may be roughly divided into three classes:—

I.—Those which require no special preparation, but may be placed directly on a slide, protected by a cover glass.

II.—Those which require to be stained in order to bring out the details of their structure.

III.—Those which require to be "cut," being too large to be at once placed under the microscope. The vast majority of specimens belong to the latter class, and for their preparation some form of microtome is necessary. Of these instruments there is a great variety, their prices ranging from a few shillings to several pounds. One of the most convenient is known as "Frazer's modification of Cathcart's microtome." It can be obtained of C. Baker, 243, High Holborn, and its cost is about twenty-five shillings.

An essential feature of any microtome is that it should be firm. In this instrument this is secured by two clamps, by means of which the oak frame is fixed to a table or working bench. In the centre is a small brass plate, roughened above for the reception of the specimen, and supported below by a micrometer screw, by which it can be lowered or raised. Under the plate the tubes from an ether spray apparatus are fixed, so that the specimens may be frozen. For "embedding" a small clamp is supplied, which is fitted in the place of the plate above described. On either side of this central fixture are two wooden supports parallel to each other, and about four inches long, on the top of which two thick slips of glass are fixed; upon these the cutting instrument runs smoothly. With the microtome is sold a large chisel-shaped knife, set in a wooden handle; this answers admirably instead of a razor. The use of the microtome will be described in another article.

Swift's microtome, consisting of a circular disc of glass, supported on a column which is clamped to the table, is also a first-class instrument for frozen specimens. The razor is fixed in a tripod stand, known as a "plough," which is passed to and fro over the specimen; after each cut the edge of the razor is lowered by means of a micrometer screw.

Besides the microscope and microtome, the following simple instruments and appliances are necessary for the work which it is proposed to describe in these articles.

Glass slides and cover glasses:—

The microscopic slides are always cut of uniform and convenient size; in this country three inches by one inch. The most useful shape for the cover glasses is a square, with seven-eighths of an inch side. The thickness known as "number 2" is preferable, being easily cleaned without breaking, and sufficiently thin for use with the oil immersion.

About half a dozen fine sewing needles mounted in wooden handles will be required. These must be kept free from rust and with unblunted points. In addition to these a pair of fine scissors, and two pairs of broad-bladed forceps with firm hinges should be procured; also a couple of section lifters. For containing stains, small glass capsules or watch glasses with ground under surfaces are necessary. To complete the list of appliances the following should be mentioned: a scalpel, two small glass funnels, some filter paper, a filter stand, a couple of glass rods, labels for slides, and soft cloth or duster.

We need not here give a tabulated list of the stains and reagents required, as we leave the reader to select those which his particular form of work would suggest, and we now pass directly to the preparation of microscopical objects.

It is extremely difficult to cut sections of specimens freshly removed from the body. Small portions may indeed be teased out by needles, or perhaps small sections cut free-hand with a razor, or by means of a Valentine's knife, but both these methods require more practice than one would care to give for such poor results. It is far more satisfactory, therefore, to prepare the specimens in such a way as to yield a sufficiently firm consistence that thin and large sections may be cut.

There are several processes for "hardening" the material which it is desired to examine, but only the two most important will be considered here.

A few preliminary remarks are, however, necessary as regards the selection of the portions to be examined. The material should be obtained as soon after death as possible; for if decomposition has set in the sections will not take up the stain properly, and thus many important characteristics are lost.

In choosing the parts of a diseased organ, the liver for example, to be hardened, it is always best to take a fragment from near the surface so as to include the capsule, as this gives great aid in recognizing the minute anatomy of the organ. Again, in preparing specimens of the intestine or stomach, small portions should be cut off transversely to the axis of the viscus, and about a quarter of an inch in thickness, so as to show the condition of its various coats. When investigating a morbid growth in any organ at least two bits should be retained; one being taken from the centre of the tumour, and one from its circumference, so as to include a portion of the organ as well as of the growth. The size of the pieces should be about quarter of an inch in length and breadth, and not quite as deep. They should be removed by means of a sharp scalpel, the cuts being cleanly made so as to avoid any tearing, and when completely freed, should be lifted up on the knife and dropped into a small open-mouthed bottle.

Undoubtedly the most convenient hardening fluid is absolute alcohol. It is suitable for all tissues except those to be presently named; it does not require to be changed, is very rapid in its action, and does not over-harden. Its chief disadvantage is that by reason of the rapidity of its action the surface of the specimen soon becomes so hard that the reagent cannot permeate the deeper portions, and consequently only very small pieces can be used.

The specimens are placed directly in the alcohol as soon as they are removed from the body, and are ready for cutting in from two to four days. This spirit is especially useful for the preparation of bacteriological work. As stated above, this reagent is not suitable for all tissues. The exceptions are: the central nervous system, the eye, and other complex tissues which do not contract regularly.

Owing to its solvent power on fat also, specimens intended to demonstrate fatty changes cannot be hardened by this fluid.

For these exceptions "Müller's Fluid" is the best hardening reagent. It has the following composition: Bichromate of potash, two parts; sodium sulphate, one part; water, 100 parts.

This is the material par excellence for the preparation of the brain and spinal cord, not for the mere process of hardening only, but as an essential factor in the best modern processes of staining sections of these parts, *i.e.*, those of Pal and Weigert. Its action is very slow, lasting over a period of four or five weeks. On the other hand, specimens of any size may be prepared by its means, even whole brains; but these must be allowed to remain in the solution for several months. In hardening by this fluid the selected parts are placed in not less than twenty times their bulk of the liquid. After twenty-four hours it will have become turbid, and must then be poured off, fresh fluid being put in its place. This must be again changed on the third and sixth days, and every week subsequently until the sixth, when the specimens should be ready for cutting. They should not be allowed to remain in the reagent too long, as they are apt to become over-hardened and brittle. At the end of about six weeks, therefore, they should be removed and thoroughly washed in water for several hours, in order to get rid of the chrome salts, after which they are transferred to methylated spirit, and can remain there until required.

Müller's Fluid must not be used when it is intended to search for micro-organisms or the demonstration of amyloid deposits, as its presence interferes with micro-chemical reactions. In order to keep the hardening fluid free from moulds a small piece of camphor should be allowed to float in it. (*To be continued.*)

